# Disparities in County-Level Vulnerability to Cardiovascular Mortality Associated With Extreme Heat Exposure

**DOI:** 10.1016/j.jacadv.2026.102590

**Published:** 2026-02-23

**Authors:** Arion Yu, Weichuan Dong, Sanjay Rajagopalan, Khurram Nasir, Sadeer Al-Kindi

**Affiliations:** a School of Medicine, Texas A&M University, Bryan, Texas, USA; b Department of Cardiology, Houston Methodist, Houston, Texas, USA; c Center for Health & Nature, Houston, Texas, USA; d Harrington Heart & Vascular Institute, University Hospitals, Cleveland, Ohio, USA

**Keywords:** cardiovascular mortality, heat, vulnerability

**What is the clinical question being addressed?**

What are the risk factors that make certain counties more susceptible to heat-related cardiovascular mortality (CVM)?

**What is the main finding?**

Sixty-eight percent of the variation in extreme heat-related CVM can be explained by county social and environmental factors, including percentage of minoritized communities, median home age, and number of mobile homes.

Global climate change related to greenhouse gas emissions has led to a rise in the intensity and frequency of extreme heat events in recent years.^1^ Excess heat exposure has been associated with increased cardiovascular strain and mortality via various physiologic mechanisms (eg, dehydration, increased cardiac output, etc).^1^ Vulnerability to heat-related cardiovascular mortality (CVM) varies widely across U.S. counties, likely reflecting differences in demographic, socioeconomic, and housing characteristics. We sought to identify county-level factors associated with heightened sensitivity to extreme heat.

## Methods

We conducted a retrospective county-level cohort analysis of 2,101 U.S. counties from 2008 to 2019. Because no individual-level data were used, Institutional Review Board approval was not required. Monthly counts of extreme heat days (heat index ≥90 °F) during May through September were derived using daily temperature and relative humidity data from National Aeronautics and Space Administration (NASA’s) Prediction of Worldwide Energy Resources (POWER), with county exposures assigned using centroid-based linkage. County-level cardiovascular deaths for the same months were obtained from the National Center for Health Statistics multiple cause-of-death files, defining CVM by International Classification of Diseases-10 codes I00–I99; death counts <10 were excluded for privacy. County-level demographic, socioeconomic, housing, and health care characteristics were obtained from the American Community Survey, County Health Rankings & Roadmaps, Area Health Resource Files, and U.S. Census data, with counties stratified by U.S. region and Rural–Urban Continuum Codes.

Excess CVM attributable to extreme heat was estimated using Poisson fixed-effects regression as the difference between observed deaths and predicted deaths under a counterfactual scenario with no extreme heat days.^2^ Random forest models with 10-fold cross-validation were then used to predict excess CVM per 1,000 CV deaths ([CV deaths modeled with observed extreme heat days – CV death modeled with 0 extreme heat days]/CV deaths modeled with 0 extreme heat days). Model performance was evaluated using R^2^ and root mean squared error, with variable importance assessed using the Boruta algorithm. All analyses were conducted in R version 4.5.1.

## Results

Among 2,101 U.S. counties, there were 3,148,880 total CV deaths, of which 19,404 were attributed to extreme heat, corresponding to 6.2 per 1,000 cardiovascular deaths. The proportion of county-level excess CV deaths ranged from 0 to 22 per 1,000 cardiovascular deaths. Mean excess deaths per 1,000 cardiovascular deaths were higher in urban counties (6.4) than in rural counties (5.5) and were highest in the South (10.9) and lowest in the Northeast (1.4). Substantial variability was observed across counties, particularly in the West.

**Figure 1:**
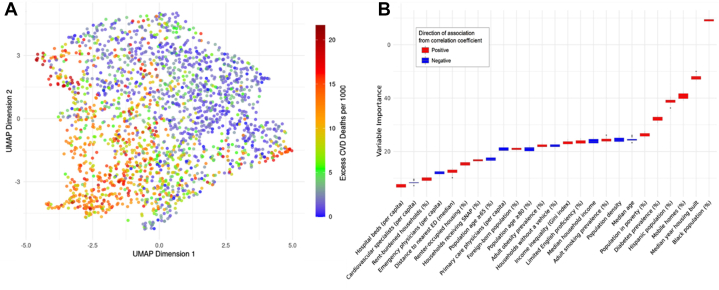
County-Level Predictors and County Clustering of Excess Cardiovascular Deaths (A) UMAP visualization showing counties positioned according to similarity among various social, demographic, housing, and health factors. Counties with similar profiles are clustered together. Color intensity reflects excess CVD deaths due to extreme heat exposure per 1,000 people, with higher values clustered together indicating shared vulnerability among counties with similar socioenvironmental profiles. (B) Boruta relative importance plot of risk factors in predicting proportion of CVM attributed to extreme heat. CVD = cardiovascular deaths; CVM = cardiovascular mortality; ED = emergency department; SNAP = Supplemental Nutrition Assistance Program; UMAP = Uniform Manifold Approximation and Projection.

Using Uniform Manifold Approximation and Projection, we visualized patterns of similarity across U.S. counties based on demographic, housing, and health variables. The representation showed a graded difference in the outcomes (Figure 1A), suggesting that socioenvironmental factors can explain county-level vulnerability to extreme heat-related CVM.

The optimal random forest model explained 68% of the variance (R^2^ = 0.68) in heat-attributed CVM with a root mean squared error of 3 deaths per 1000 CV deaths. The Boruta algorithm identified several demographic and housing factors as the top predictors of CVM due to extreme heat (Figure 1B). Of these, counties with a higher percentage of Black and Hispanic residents, mobile homes, and median year of home construction demonstrated a higher number of heat-related mortality. Other predictors included diabetes prevalence, percentage of population below the poverty line, and increased population density.

## Discussion

This study demonstrates that vulnerability to these mechanisms may be amplified by underlying socioenvironmental conditions. Our findings demonstrate that 68% of county-level vulnerability measure is associated with a range of social, demographic, housing, and health factors. Percentage of residents from minoritized communities emerged as the most important factors for vulnerability, highlighting environmental justice factors. Additional housing-related factors, such as mobile homes and house age, were associated with disproportionate impact. These results are aligned with prior work demonstrating racial and socioeconomic disparities in CV health outcomes.^3^^,^^4^ Further research could investigate the role of socioeconomic disparities in access to air conditioning and the type of insulation in housing units especially in marginalized communities despite advances in cooling technology.

Our findings point to the importance of equity-centered adaptation strategies. Targeted investments in heat-resilient infrastructure, subsidies for cooling technologies, and strengthening of health care access in high-risk counties may help mitigate disparities. In addition, early warning systems and community-based interventions like recognizing signs of heat stress can be tailored to vulnerable populations.^5^ Patients in high-risk counties could be advised to employ low-cost cooling strategies such as electric fans and to increase fluid intake to avoid dehydration. Addressing these inequities is critical as extreme heat events become more frequent with advancing climate change.

This observational study cannot establish causality and may be subject to time-varying confounding despite fixed-effects modeling. Heat exposure estimates relied on centroid-based NASA POWER data, and CVM was identified using death certificates, which may be misclassified. Exclusion of counties with fewer than 10 deaths may bias results toward more populated areas. Several covariates were derived from self-reported survey data and may be prone to reporting bias.

## Conclusions

Heat-related CVM was predicted with county characteristics with percentage of minoritized residents, median house age, and mobile homes being the most predictive factors. These findings can inform targeted public health and infrastructure interventions as extreme heat increases.

## Funding support and author disclosures

The authors have reported that they have no relationships relevant to the contents of this paper to disclose.

## References

[1] Khraishah H., Alahmad B., Ostergard R.L. (2022). Climate change and cardiovascular disease: implications for global health. Nat Rev Cardiol.

[2] Khatana S.A.M., Eberly L.A., Nathan A.S., Groeneveld P.W. (2023). Projected change in the burden of excess cardiovascular deaths associated with extreme heat by midcentury (2036–2065) in the contiguous United States. Circulation.

[3] Dong W., Motairek I., Nasir K. (2023). Risk factors and geographic disparities in premature cardiovascular mortality in US counties: a machine learning approach. Sci Rep.

[4] Salerno P.R.V.O., Motairek I., Dong W. (Published online April 3, 2024). County-Level Socio-Environmental factors associated with stroke mortality in the United States: a cross-sectional study. Angiology.

[5] Rajagopalan S., Vergara-Martel A., Zhong J. (2024). The urban environment and cardiometabolic health. Circulation.

